# Ultrasound-assisted electrodeposition of indium from HCl stripped indium-loaded organic phase solutions by EQCM: Electrochemical behavior and nucleation mechanisms

**DOI:** 10.1016/j.ultsonch.2025.107410

**Published:** 2025-05-30

**Authors:** Shiju Li, Haibei Wang, Shengdong Wang, Feng Xie, Xudong Sun

**Affiliations:** a School of Metallurgy, Northeastern University, Shenyang 110819, China; b BGRIMM Technology Group, Beijing 100160, China

**Keywords:** Stripping solution, Indium, Electrodeposition, Ultrasound-assisted, EQCM

## Abstract

In order to reduce the duration of the recovery process for indium from zinc oxide dust, an electrodeposition technique was employed, utilizing a hydrochloric acid stripping solution as the electrolyte. A study of the cyclic voltammetry-EQCM of conventionally electrodeposited indium and of ultrasound-assisted electrodeposition of indium was undertaken. The valence transition of conventionally electrodeposited indium was found to be In^2+^-In^+^, In^3+^-In; that of ultrasound-assisted electrodeposition of indium is In^2+^-In^+^-In, In^3+^-In. Furthermore, the Scharifker and Hills model underwent modification due to the negative values of the timed amperage curve currents, which resulted in the dimensionless processed timed amperage curve (j/j_max_)^2^-(t/t_max_) conforming to the specification. Concurrently, it was determined that ultrasound-assisted electrodeposition has the capacity to impede the precipitation of hydrogen, chlorine, and trivalent arsenic on the cathode deposition. The chronoamperometric study demonstrated that conventional electrodeposition exhibited transient nucleation at deposition potentials of −0.75 V, −0.78 V, −0.80 V, and −0.82 V. Conversely, ultrasound-assisted electrodeposition manifested transient nucleation at deposition potentials of −0.75 V, −0.78 V, and −0.82 V, along with gradual nucleation at the −0.80 V potential. Furthermore, at the same electrodeposition potential, ultrasound-assisted electrodeposition resulted in greater nucleation density values (N) than conventional electrodeposition. Furthermore, ultrasound-assisted electrodeposition has been shown to enhance current efficiency. In addition, scanning electron microscopy with energy-dispersive spectroscopy (SEM-EDS) analysis has revealed that the indium deposited on the cathode exhibits high purity. Moreover, the surface morphology of the indium layer deposited by conventional electrodeposition is characterized by flocculent structures, while the indium layer deposited by ultrasound-assisted electrodeposition demonstrates a spherical morphology. This provides a novel concept for the large-scale production of indium sponge.

## 1 Introduction

Indium, a rare metal, is present in the earth's crust and oceans at concentrations of approximately 0.05 ppm and 0.072 ppm, respectively [1]. It is primarily recovered from by-products of zinc metal smelting, including zinc oxide dust and zinc slag [2,3]. Specifically, the indium content of zinc oxide dust from the roasting of zinc concentrates is typically in the range of 0.1 % to 1.0 %, and it is a primary raw material in the recovery of indium. Indium, a rare and essential metal, is employed in numerous high-tech fields, including display technology, semiconductor manufacturing, and solar photovoltaic cells [4]. Consequently, numerous countries have identified indium as a pivotal strategic metal, leading to an augmentation in their reserves of this element [5].

At present, the principal processes for recovering indium are leaching, extraction, stripping, and zinc plate replacement of the stripping solution to obtain indium sponge [6]. However, utilizing zinc or aluminums plates to substitute for the stripping solution in the extraction of indium sponge results in the consumption of these metals, consequently leading to a waste of resources. Concurrently, the replacement reaction generates arsine, a highly toxic substance that poses a significant threat to the environment and human health [7]. Furthermore, the indium sponge obtained must undergo electrolytic refinement once more to yield indium concentrate, a process that undeniably prolongs the indium production process and is economically unviable. Consequently, the development of a short-flow process for the recovery of indium is of particular importance.

Direct electrodeposition of indium from the stripping solution is a viable option; however, given the strong acidic nature of the stripping solution, a significant amount of hydrogen precipitation (OER) is inevitable, resulting in a decline in current efficiency. This, in turn, leads to an increase in energy consumption and the embrittlement of the deposited layer due to hydrogen. Moreover, at the anode, the process is accompanied by the precipitation of chlorine gas, a toxic gas that renders the production environment of electrodeposited indium unsafe. In order to circumvent HER, a significant number of researchers have attempted to electrolyze indium in non-aqueous solutions, including ionic liquids, molten salts, and deep eutectic solvents [[8], [9], [10], [11], [12]]. However, this process is costly and challenging to scale up for industrial application.

In recent years, ultrasound has been extensively utilized in industrial settings, such as leaching and welding, due to its cavitation, mechanical and thermal effects [[13], [14], [15]]. Furthermore, some researchers have applied ultrasound to electrodeposited composite coatings and achieved superior outcomes [[16], [17], [18], [19]]. In the future, the potential for ultrasound to be applied to more fields is significant, including electrodeposition of metals from aqueous solutions, nanomaterial synthesis and electrolytic refining of metals.

The Electrochemical Quartz Crystal Microbalance (EQCM) is a highly versatile instrument that finds application in a wide range of fields, including metal electrodeposition [[20], [21], [22]], electrochemical synthesis [23,24], metal corrosion [[25], [26], [27], [28]], and other fields related to interfacial phenomena. It has the capacity to monitor the mass change in the electrochemical process in real time at the nanogram level, which facilitates the study of the electrochemical behavior of the electrodeposition process in the stripping solution and the valence transition and nucleation mechanism of indium. In the study conducted by Hu et al. (2022), the electrochemical behavior of indium electrodeposited in acidic aqueous solution was investigated through the utilization of an electrochemical quartz crystal microbalance (EQCM). The findings of this study demonstrated that the nucleation mechanism of indium on Pt electrodes is characterized by progressive nucleation [29].

In this study, an electrochemical quartz crystal microbalance (EQCM) was employed to electrodeposit indium directly from the aqueous phase obtained by hydrochloric acid stripping of the indium-loaded organic phase, relying on ultrasound assistance. This method was compared with conventional electrodeposition. Furthermore, modifications were made to the Scharifker and Hills model. Following comprehensive research in the relevant academic literature, it is evident that this work has not been previously documented. Its novelty is therefore self-evident.

## 2 Experimental

### 2.1 Preparation of the stripping solution

The solution of hydrochloric acid stripping of the organic phase loaded with indium was prepared by subjecting the zinc oxide dust leachate to precipitation, extraction, and stripping. The method of preparation was described in detail in a series of previously published papers by this group [[30], [31], [32]]. The composition of the stripping solution is shown in Table 1.

**Table 1:** Composition of stripping solutions used in electrodeposition studies.

Element	In	Zn	Fe	Al	Cu	As
Concentration, g/L	2.022	0.261	0.290	0.00605	0.00014	0.000056

### 2.2 Experimental equipment and methods

The ultrasonic equipment under scrutiny in this study is manufactured by Wuxi Yuansheng Intelligent Technology Co., Ltd. The model of the equipment is designated as the LS-1200B. The resonant frequency of the equipment is 20 kHz, and the power output ranges from 5 to 800 W. The probe material is TC4 titanium alloy, and the probe size is 2 mm in diameter. The experimental investigation entailed the assessment of the electrodeposition of indium, utilizing a chronoamperometric current curve approach. This was achieved through the implementation of a quartz crystal microbalance (EQCM, CHI400C) manufactured by Shanghai Chenhua Instruments Co. The aforementioned EQCM was employed to conduct a cyclic voltammetry analysis. The experiment employed a three-electrode system, comprising a working electrode, which was a crystal electrode, a counter electrode, which was a platinum wire, and a reference electrode, which was a standard silver/silver chloride electrode (Ag/AgCl, E_0_=+0.2224 V, 25 °C). The EQCM incorporates a bespoke electrolytic cell comprising three circular pieces of PTFE. The crystal electrode, which is mounted at the base of the cell, is plated with titanium and gold, and has an area of 0.196 cm^2^. The reference frequency of f_0_ is 7.995 MHz Furthermore, a series of constant potential electrodeposition experiments were conducted within a 50 ml quartz electrolyte, utilizing a three-electrode system. This comprised a platinum sheet employed as both the working and counter electrodes, with a standard silver/silver chloride electrode designated for the reference electrode (Ag/AgCl, E_0_ = +0.2224 V, 25 °C). Prior to experimentation, the electrodes were meticulously cleaned with a solution of ultrapure water and ethanol, followed by drying with a hair dryer. This process was repeated at the commencement and conclusion of each experiment to ensure the maintenance of optimal conditions. The relationship between the change in electrode frequency (Δf) and the change in mass (Δm) is described by the Sauerbrey equation:

(1) Δm=K×Δf

Where: Δm is the mass change of the quartz crystal oscillator; K is the sensitivity coefficient of the quartz wafer, which indicates the amount of change in the electrode mass for each change in the crystal frequency of 1 Hz, and the value of K in this experiment is 1.34 ng⋅Hz^−1^, Δf is the frequency change of the quartz crystal oscillator.

### 2.3 Theory of electrodeposition

The electrodeposition of indium can be considered an instance of an electrochemical reaction, within which redox reactions and the transfer of electrons occur at the electrodes. This results in a change in the mass of the electrodes, and thus, in accordance with Faraday's law [33]:

(2) $$\Delta m=\frac{M}{n\times F}\times \Delta Q$$

Where Δm is the mass change of electrodeposited indium, M is the molar mass of indium deposited on the electrode, n is the number of electrons transferred, F is the Faraday's constant (96485C/mol), and ΔQ is the amount of charge transferred on the electrode, transforming the equation (2) leads to equation (3):

(3) $$\frac{M}{n}=\frac{\Delta m}{\Delta Q}\times F$$

The actual M/n value can be calculated from the experimentally measured value Δm/ΔQ, and the theoretical M/n value can be calculated from the electrochemical reaction equation, and then, comparing the actual value and theoretical value, if the difference between these two values is not too big, then it can be judged whether or not this electrochemical reaction occurs.

The current efficiency of electrodeposited indium can be calculated by equation (4):

(4) $$\eta =\frac{mnF}{QM}\times 100\%$$

Where: η is the current efficiency, %; m is the mass change of the working electrode before and after electrodeposition, g; n is the number of transferred electrons; F is the Faraday constant 96485C/mol; Q is the total charge consumed by electrodeposition; M is the relative atomic mass of indium, M(In) = 114 g/mol.

### 2.4 Characterization

**Fig. 1:**
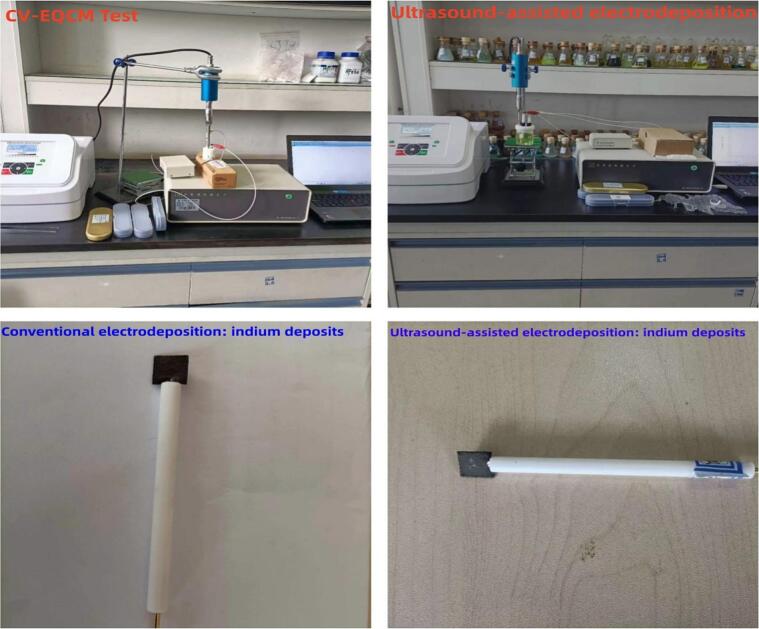
Practical experiments of conventional electrodeposition of indium and ultrasound assisted electrodeposition of indium.

The UV–vis (Model: UH4150) absorption spectra of the stripping solution and the electrodeposition residual solution were examined. Following the constant potential electrodeposition experiments, the working electrode was rinsed with deionized water and dried for 12 h. Due to the minimal amount of indium deposited on the Pt electrode, a decision was taken to excise half of it, with the deposited layer then being tested directly on the Pt electrode by XRD and SEM-EDS. The X-ray diffractometer model is designated as “Ultima IV”, whilst the Scanning Electron Microscope (SEM) model is the “Quanta 600″ and the Energy Spectrometer (EDS) model is the ”Genesis 7000″. As demonstrated in Fig. 1, the experimental procedures of conventional electrodeposition and ultrasound-assisted electrodeposition of indium are illustrated.

## 3 Results and discussion

### 3.1 Cyclic voltammetry-EQCM analysis

#### 3.1.1 Conventional

**Fig. 2:**
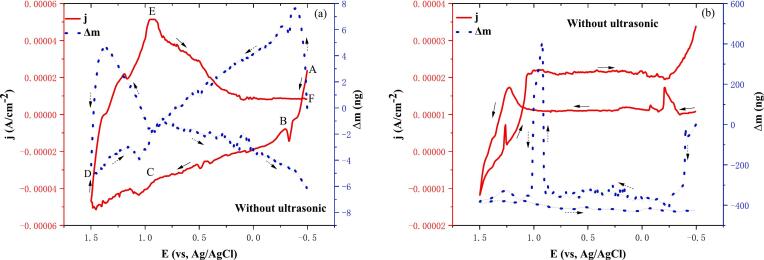
Cyclic voltammetry-EQCM curves for conventional electrodeposition (a. Stripping solution; b. 2 mol/L hydrochloric acid. Temperature 25℃, scan rate: 0.05 V/s).

As demonstrated in Fig. 2a, the conventional cyclic voltammetry (CV)-EQCM curves of stripping solutions are illustrated, as tested using an electrochemical quartz crystal microbalance (EQCM). In order to facilitate the analysis, the CV curves were divided into regions (A-B, B-C, C-D, D-E, E-F) depending on the potential. Furthermore, a 2 mol/L blank HCl solution was employed as a control, as demonstrated in Fig. 2b. Concurrently, the theoretical M/n values of the electrochemical reactions were calculated using equations (1), (2), and (3). Subsequently, the actual M/n values were derived from experimental measurements, which are shown in Table 2.

**Table 2:** Experimental values of M/n obtained from EQCM and CV in Fig. 2a.

Region	Potential region (V)	Possible chemical reaction	Theoretical values (g/mol)	Experimental values (g/mol)
A-B	−0.5 to −0.31	Eq. (5)	114.8	324.49
		Eq. (6)	38.27	28.12

B-C	−0.31 to 0.91	Eq. (7)	1	−65.83
		Eq. (8)	35.97	−13.08
		Eq. (9)	31.75	−3.79
		Eq. (10)	63.5	−1.44
		Eq. (11)	16	−0.19
		Eq. (12)	56	0.52

C-D	0.91 to 1.48	Eq. (13)	49.75	2.06
		Eq. (14)	27.83	1.54
		Eq. (15)	35.5	1.62
		Eq. (16)	12.44	1.65
		Eq. (17)	13.92	1.66

D-E	1.48 to 0.91	Eq. (18)	34.25	2.19
		Eq. (19)	41.75	−0.89

E-F	0.91 to -0.50	Eq. (20)	38.27	−6.11
		Eq. (21)	114.8	−62.60

Within the potential interval A-B (−0.50 V to −0.31 V), a decline in current density and an augmentation in mass were observed, thereby substantiating the electrodeposition of indium. Furthermore, two conspicuous reduction peaks were identified at −0.407 V and −0.329 V, which may be associated with the (In^2+^/In^+^) and (In^3+^/In) peaks, respectively. Furthermore, as demonstrated in Fig. 2b, the mass curve exhibits a negative downward trend within the potential interval of −0.50 V to −0.31 V, thereby indicating that no metals undergoes electrodeposition. This outcome serves to validate the precision of the EQCM measurement. The electrochemical reaction equations are (5) and (6), respectively. As demonstrated in Table 2, the theoretical and actual M/n values of equations (5), (6) are closely aligned, suggesting a potential for reaction.

(5) $${In}^{2+}+e^{-}={In}^{+}\;\;E_{0}=-0.40V\;vs\;SHE$$

(6) $${In}^{3+}+3e^{-}=In\;\;E_{0}=-0.3382V\;vs\;SHE$$

In the B-C potential interval (−0.31 V to 0.91 V), it can be seen from Fig. 2a that the current density and mass of the CV-EQCM curves decreased, and six distinct reduction peaks were observed at 0.067 V, 0.241 V, 0.397 V, 0.493 V, 0.661 V, and 0.775 V, which may correspond to H^+^/H_2_, As^3+^/As, Cu^2+^/Cu, O_2_/O^-^, Fe^3+^/Fe^2+^ peaks respectively, and the electrochemical reaction equations are shown in (7)-(12). Table 2 shows that the theoretical and actual M/n values for equations (7), (8) are close to each other, therefore, the reaction may occur. Equations (9)-(12) have a large difference between the theoretical M/n value and the actual M/n value, therefore the reaction may not occur.

(7) $$\;2H^{+}+2e^{-}=H_{2}\;\;\;E_{0}=0V\;vs\;SHE$$

(8) $$HAsO_{2}+3H^{+}+3e^{-}=As+2H_{2}O\;\;E_{0}=0.248V\;vs\;SHE$$

(9) $${Cu}^{2+}+2e^{-}=Cu\;\;E_{0}=0.342V\;vs\;SHE$$

(10) $${Cu}^{+}+e^{-}=Cu\;\;E_{0}=0.521V\;vs\;SHE$$

(11) $$O_{2}+{2H}^{+}+{2e}^{-}=H_{2}O_{2}\;\;E_{0}=0.695V\;vs\;SHE$$

(12) $${Fe}^{3+}+e^{-}={Fe}^{2+}\;\;E_{0}=0.771V\;vs\;SHE$$

Within the C-D potential interval (0.91 V to 1.48 V), as illustrated in Fig. 2a, it is evident that the current density of the CV-EQCM curve experiences a decline, while the mass curve exhibits an initial rise followed by a decrease. This observation serves as a testament to the predominant occurrence of electrochemical reactions involving the interconversion of chlorine elements within this specific potential interval. Five oxidation peaks were identified at 1.057 V, 1.237 V, 1.357 V, 1.399 V, and 1.459 V, respectively. These peaks may correspond to the five oxidation peaks of ClO_4_^-^/ClO_3_^-^, ClO_3_^-^/HClO_2_, Cl_2_/Cl^-^, and ClO_4_^-^/Cl_2_, and the electrochemical reaction equations as (13) through (17) are shown. As demonstrated in Table 2, a significant disparity is evident between the theoretical and actual M/n values of equations (13), (17). However, the actual M/n values are positive, indicating that the reaction is probable.

(13) $$ClO_{4}^{-}+2H^{+}+2e^{-}={ClO}_{3}^{-}+H_{2}O\;\;E_{0}=1.189V\;vs\;SHE$$

(14) $${ClO}_{3}^{-}+3H^{+}+2e^{-}={HClO}_{2}+H_{2}O\;\;E_{0}=1.214V\;vs\;SHE$$

(15) $${Cl}_{2}+2e^{-}=2{Cl}^{-}\;\;E_{0}=1.358V\;vs\;SHE$$

(16) $${2ClO}_{4}^{-}+16H^{+}+14e^{-}={Cl}_{2}+8H_{2}O\;\;E_{0}=1.39V\;vs\;SHE$$

(17) $${ClO}_{3}^{-}+6H^{+}+6e^{-}={Cl}^{-}+3H_{2}O\;\;E_{0}=1.457V\;vs\;SHE$$

In the D-E potential interval (1.48 V to 0.91 V), as illustrated in Fig. 2a, it is evident that the current density of the CV-EQCM curve exhibited an increase, while the mass curve demonstrated a negative trend. This observation substantiates the hypothesis that the oxidation reaction predominantly occurred within this specific potential interval. Two oxidation peaks were identified at 1.187 V and 0.941 V, which may correspond to the two oxidation peaks of ClO_3_^-^/ClO_4_^-^ and HClO_2_/ClO_3_^-^ respectively. The electrochemical reaction equations are shown in (17) and (18). As demonstrated in Table 2, the substantial discrepancy between the theoretical and actual M/n values of equation (18) suggests a low probability of reaction occurrence. Conversely, the positive actual M/n value of equation (19) indicates a high probability of reaction occurrence.

(18) $${HClO}_{2}+H_{2}O-2e^{-}={ClO}_{3}^{-}+2H^{+}\;\;E_{0}=1.214V\;vs\;SHE$$

(19) $${ClO}_{3}^{-}+H_{2}O-2e^{-}=ClO_{4}^{-}+2H^{+}\;\;E_{0}=1.189V\;vs\;SHE$$

Within the E-F potential interval (0.91 V to −0.50 V), a decline in the current density of the CV-EQCM curve and a negative mass curve are evident in Fig. 2a, thereby substantiating the premise that the oxidation reaction predominantly transpires within this specific potential interval. Two distinct oxidation peaks were observed at 0.395 V and 0.131 V, respectively, which may correspond to the two oxidation peaks of In/In^3+^ and In/In^+^. The electrochemical reaction equations are shown in (19) and (20).

(20) $$In-3e^{-}=In\;\;E_{0}=0.3382V\;vs\;SHE$$

(21) $$In-e^{-}={In}^{+}\;\;E_{0}=0.14V\;vs\;SHE$$

#### 3.1.2 Ultrasonic

##### 3.1.2.1 Effect of different ultrasonic powers on cyclic voltammetry

**Fig. 3:**
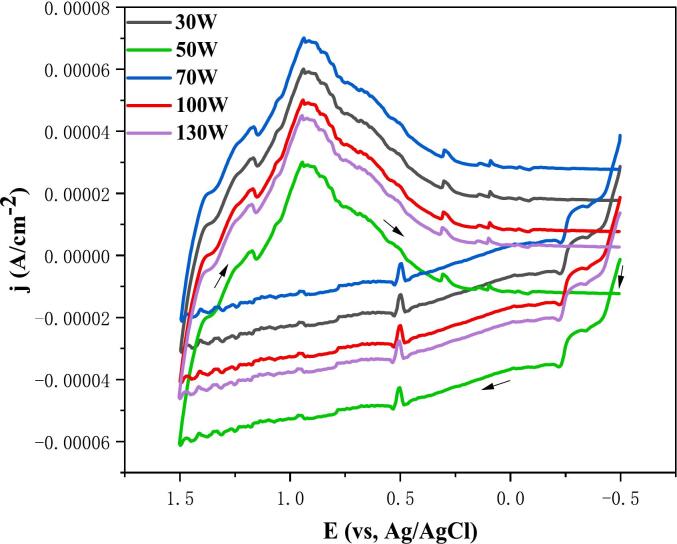
Effect of different ultrasonic power on cyclic voltammetry (scan rate: 0.05 V/s).

As demonstrated in Fig. 3, the impact of varying ultrasonic power on cyclic voltammetry is illustrated. It is evident from the figure that a change in ultrasonic power results in a corresponding alteration in peak current density. However, the influence on oxidation and reduction potential is minimal, thereby substantiating the notion that ultrasound can induce side reactions within the electrolyte. Consequently, insufficient or excessive ultrasonic power is disadvantageous to the electrodeposition of indium. In the subsequent CV-EQCM test, an ultrasonic power of 100 W was selected.

##### 3.1.2.2 Ultrasonic-assisted CV-EQCM

**Fig. 4:**
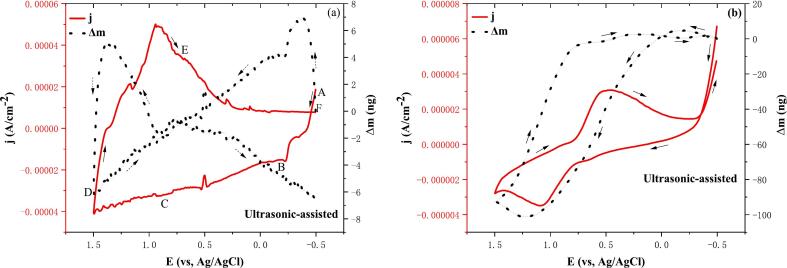
Cyclic voltammetry-EQCM curves for ultrasonic-assisted electrodeposition (a. Stripping solution; b. 2 mol/L hydrochloric acid. Ultrasonic power 100 W, scan rate: 0.05 V/s).

As illustrated in Fig. 4a, the cyclic voltammetry-EQCM curves of the ultrasound-assisted stripping solution are presented. In order to facilitate analysis, the CV curves have been divided into a number of potential intervals, including A-B (−0.50 V to −0.20 V), B-C (−0.20 V to 0.91 V), C-D (0.91 V to 1.48 V), D-E (1.48 V to 0.73 V), and E-F (0.73 V to −0.50 V). Concurrently, a blank 2 mol/L hydrochloric acid solution was tested under ultrasound assistance to serve as a reference point, as illustrated in Fig. 4b. The theoretical and actual M/n values for the electrochemical reaction equations are listed in Table 3.

**Table 3:** Experimental values of M/n obtained from EQCM and CV in Fig. 4a.

Region	Potential region (V)	Possible chemical reaction	Theoretical values (g/mol)	Experimental values (g/mol)
A-B	−0.5 to −0.20	Eq. (22)	114.8	295.50
		Eq. (23)	38.27	113.96
		Eq. (24)	114.80	28.75

B-C	−0.20 to 0.91	Eq. (25)	32.50	0.14
		Eq. (26)	65.00	0.84
		Eq. (27)	56.00	0.99

C-D	0.91 to 1.48	Eq. (28)	41.75	2.19
		Eq. (29)	27.80	2.33
		Eq. (30)	67.50	2.34
		Eq. (31)	12.44	2.37
		Eq. (32)	13.92	2.53

D-E	1.48 to 0.73	Eq. (33)	67.50	2.68
		Eq. (34)	34.25	−0.89

E-F	0.73 to −0.50	Eq. (35)	38.27	−11.91
		Eq. (36)	114.80	−48.67

Within the potential interval A-B (−0.50 V to −0.20 V), as illustrated in Fig. 4a, the current density of the CV-EQCM curve decreases while the mass curve increases. This finding suggests that the electrodeposition of metals occurs within this potential interval. Furthermore, three distinct reduction peaks were identified at −0.407 V, −0.347 V, and −0.227 V, which correspond to In^2+^/In^+^, In^3+^/In, and In^+^/In peaks, respectively. These peaks can be indicative of the electrochemical reaction equations (22), (23), (24). As demonstrated in Table 3, the theoretical M/n values of equations (22)-(24) exhibit a minor discrepancy from their actual values, which are positive. This observation suggests the potential for these reactions to occur. Furthermore, the valence state transition of indium by ultrasound-assisted electrodeposition was found to be In^2+^-In^+^-In, In^3+^-In.

(22) $${In}^{2+}+e^{-}={In}^{+}\;\;E_{0}=-0.40V\;vs\;SHE$$

(23) $${In}^{3+}+3e^{-}=In\;\;E_{0}=-0.3382V\;vs\;SHE$$

(24) $${In}^{+}+e^{-}=In\;\;E_{0}=-0.14V\;vs\;SHE$$

Within the potential interval B-C (−0.20 V to 0.91 V), as illustrated in Fig. 4a, the current density of the CV-EQCM curve and the mass curve both decrease, and three distinct reduction peaks are observed at 0.481 V, 0.529 V, and 0.727 V, which may correspond to the Cu^2+^/Cu, Cu^+^/Cu, and Fe^3+^/Fe^2+^ peaks, respectively. It is interesting to note that no obvious reduction peak of precipitated hydrogen was observed in the B-C potential interval. This finding indicates that ultrasound-induced microfluidic flow accelerates the diffusion of hydrogen ions and reduces the concentration of hydrogen ions on the electrode surface, thereby reducing the precipitation of hydrogen. This phenomenon may be consistent with the electrochemical reaction equation (25)-(27). As illustrated in Table 3, a significant disparity is evident between the theoretical and actual M/n values for equations (25)-(27). However, the observation of positive values in these equations suggests the potential for reaction to occur.

(25) $${Cu}^{2+}+2e^{-}=Cu\;\;E_{0}=0.342V\;vs\;SHE$$

(26) $${Cu}^{+}+e^{-}=Cu\;\;E_{0}=0.521V\;vs\;SHE$$

(27) $${Fe}^{3+}+e^{-}={Fe}^{2+}\;\;E_{0}=0.771V\;vs\;SHE$$

Within the potential interval C-D (0.91 V to 1.48 V), as illustrated in Fig. 4a, the current density of the CV-EQCM curve decreases, and the mass curve first rises and then decreases, with five distinct reduction peaks observed at 1.171 V, 1.249 V, 1.309 V, 1.375 V, and 1.447 V, which may correspond to the ClO_3_^-^/ClO_2_,ClO_3_^-^/HClO_2_, ClO_2_/HClO_2_,ClO_4_^-^/Cl_2_,ClO_3_^-^/Cl^-^ peaks, which may correspond to the electrochemical reaction equations (28)-(32). As illustrated in Table 3, a significant disparity is evident between the theoretical and actual M/n values for equations (28)-(32). However, it is noteworthy that these values are positive, suggesting the potential for reaction to occur.

(28) $${ClO}_{3}^{-}+2H^{+}+e^{-}={ClO}_{2}+H_{2}O\;\;E_{0}=1.152V\;vs\;SHE$$

(29) $${ClO}_{3}^{-}+3H^{+}+2e^{-}={HClO}_{2}+H_{2}O\;\;E_{0}=1.214V\;vs\;SHE$$

(30) $${ClO}_{2}+H^{+}+e^{-}={HClO}_{2}\;\;E_{0}=1.277V\;vs\;SHE$$

(31) $${2ClO}_{4}^{-}+16H^{+}+14e^{-}={Cl}_{2}+8H_{2}O\;\;E_{0}=1.39V\;vs\;SHE$$

(32) $${2ClO}_{3}^{-}+12H^{+}+10e^{-}={Cl}_{2}+6H_{2}O\;\;E_{0}=1.47V\;vs\;SHE$$

Within the D-E (1.48 V to 0.73 V) potential interval, as illustrated in Fig. 4a, the current density of the CV-EQCM curve increases and the mass curve rises yet remains consistently negative. This observation indicates that oxidation reaction occurs within this potential interval. Two discrete oxidation peaks were identified at 1.169 V and 0.941 V, which may be indicative of HClO_2_/ClO_3_^-^ and ClO_2_/ClO_3_^-^ peaks, respectively. Meanwhile, no discernible Cl^-^/Cl_2_ peaks were observed in the potential interval D-E, thereby indicating that the introduction of ultrasound could indeed inhibit the precipitation of chlorine. This phenomenon has been attributed to the cavitation of ultrasound, which results in the formation of cavitation bubbles. The energy released when these bubbles undergo rupture has been postulated to destroy chlorine vesicles on the surface of the electrodes, thereby reducing the accumulation of chlorine. This may correspond to the electrochemical reaction equations (33), (34). As illustrated in Table 3, there is a substantial discrepancy between the theoretical and actual M/n values for equation (33). However, the actual M/n value is positive, suggesting that the reaction is probable. The discrepancy between the theoretical and actual M/n values for equation (34) is substantial, yet the actual M/n value is negative, indicating a low probability of reaction occurrence.

(33) $${{ClO}_{2}+H_{2}O-e^{-}=ClO}_{3}^{-}+2H^{+}\;\;E_{0}=1.152V\;vs\;SHE$$

(34) $${{HClO}_{2}+H_{2}O-2e^{-}=ClO}_{3}^{-}+3H^{+}\;\;E_{0}=1.214V\;vs\;SHE$$

Within the E-F (0.73 V to −0.50 V) potential interval, as illustrated in Fig. 4a, the current density of the CV-EQCM curve decreases, whilst the mass curve decreases and becomes negative, indicating that metal dissolution occurs within this potential interval. Two distinct oxidation peaks were observed at 0.299 V and 0.143 V, which may correspond to the In/In^3+^ and In/In^+^ peaks, respectively. The corresponding electrochemical reaction equations are (35) and (36).

(35) $$In-3e^{-}={In}^{3+}\;\;E_{0}=0.3382V\;vs\;SHE$$

(36) $$In-e^{-}={In}^{+}\;\;E_{0}=0.14V\;vs\;SHE$$

Furthermore, the substantial discrepancies observed between the theoretical and actual values presented in Table 2, Table 3 may be attributed to various factors, including side reactions (e.g., hydrogen precipitation, oxygen precipitation, electrolyte decomposition), co-deposition, viscoelastic effects, interference from environmental factors, adsorption phenomena, and experimental manipulation errors. The aforementioned errors can be rectified by optimizing the electrolyte and controlling the experimental conditions.

### 3.2 Chronoamperometry to study the nucleation mechanism of indium

#### 3.2.1 Nucleation mechanism of conventional electrodeposited indium

**Fig. 5:**
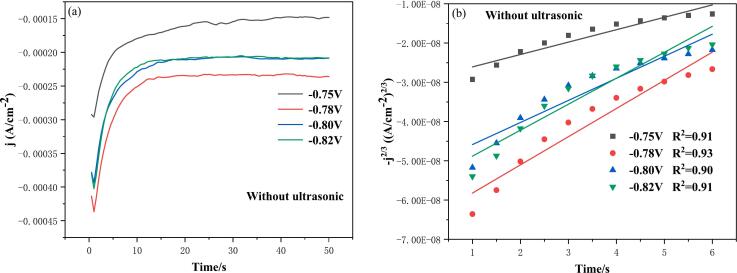
(a) Chronoamperometric curves of conventional electrodeposition of indium at different deposition potentials; (b) −j^2/3^-t plot of the rising part of the transient current; Temperature 25℃.

As illustrated in Fig. 5, the chronoamperometry curve of conventional electrodeposition of indium at varying deposition potentials, with a deposition time of 50 s, are presented.

From Fig. 5a, it can be seen that the chronoamperometric curve can be divided into three regions, (i) the current density firstly shows a decreasing trend, at the beginning of electrodeposition a bilayer may be formed, and the charging process of the bilayer consumes the current, which subsequently decreases after the charging is completed; (ii) the current density gradually increases to the maximum, which is typical of the nucleation and growth of crystals; and (iii) with the proceeding of the deposition, the surface of the electrodes is gradually covered by the covered by the deposits, the effective reaction area tends to stabilize, leading to a stabilization of the current density. Fig. 5b shows the fitting curve of the rising part of the current density to the transient time of Fig. 5a, yielding a −j^2/3^-t plot. Some researchers have demonstrated that the nucleation mode of electrodeposited metals can initially be determined by the relationship between the rising portion of the current and the time of the current transient, and that in the case of three-dimensional diffusion-controlled progressive nucleation, the −j^2/3^ of the current density is linearly related to t [34]. The R^2^ of Fig. 5b are all greater than 0.9, indicating that the −j^2/3^ of the current density has a good linear relationship with t. Therefore, it can be initially determined that the nucleation mode of conventional electrodeposited indium is progressive nucleation.

In order to accurately determine the nucleation model for the indium electrodeposition process, reference is made to the three-dimensional nucleation model proposed by Scharifker and Hills [[35], [36], [37]], as shown in equations (37)-(38):

(37) $$({\frac{j}{j_{max}})}^{2}=\frac{1.9542}{\left(\frac{t}{t_{max}}\right)}{\left\{1-\exp\left[-1.2564\left(\frac{t}{t_{max}}\right)\right]\right\}}^{2}\;\;Instantaneous\;nucleation$$

(38) $${\left(\frac{j}{j_{max}}\right)}^{2}=\frac{1.2254}{\left(\frac{t}{t_{max}}\right)}{\left\{1-\exp{\left[-2.3367\left(\frac{t}{t_{max}}\right)\right]}^{2}\right\}}^{2}\;\;Progressive\;nucleation$$

**Fig. 6:**
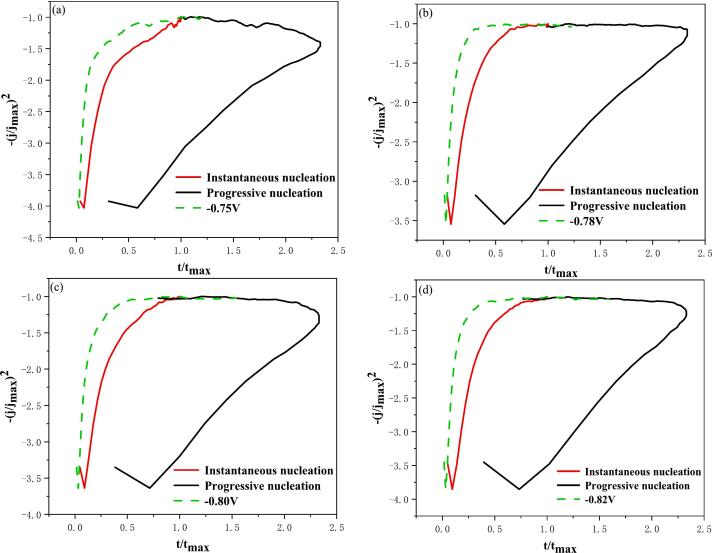
Non-dimensional-(j/j_max_)^2^ vs t/t_max_ plots at different electrodeposition potentials of conventional electrodeposition: (a) −0.75 V; (b) −0.78 V; (c) −0.80 V; (d) −0.82 V.

However, as demonstrated in Fig. 5a, the transient currents are found to be negative. Consequently, if the transient dimensionless current values obtained following the dimensionless treatment based on the aforementioned nucleation model are positive, it is imperative to correct equations (37), (38). The correction yields equations (39), (40). Subsequent to the dimensionless treatment of Fig. 5a based on equations (39), (40), Fig. 6 is obtained.

(39) $$-({\frac{j}{j_{max}})}^{2}=\frac{1.9542}{\left(\frac{t}{t_{max}}\right)}{\left\{1-\exp\left[-1.2564\left(\frac{t}{t_{max}}\right)\right]\right\}}^{2}\;\;Instantaneous\;nucleation$$

(40) $$-{\left(\frac{j}{j_{max}}\right)}^{2}=\frac{1.2254}{\left(\frac{t}{t_{max}}\right)}{\left\{1-\exp{\left[-2.3367\left(\frac{t}{t_{max}}\right)\right]}^{2}\right\}}^{2}\;\;Progressive\;nucleation$$

As demonstrated in Fig. 6, the dimensionless curves of varying deposition potentials demonstrate a closer proximity to the theoretical curve of instantaneous nucleation and a greater distance from the theoretical curve of progressive nucleation. This observation suggests that the process of conventional electrodeposition of indium can be classified as instantaneous nucleation. This phenomenon can be attributed to the stripping solution, which is formed by the process of hydrochloric acid stripping the organic phase loaded with indium. This solution has a low pH and is strongly acidic. In addition, the process of electrodeposited indium is more inclined to instantaneous nucleation under the condition of strongly acidic electrolyte.

#### 3.2.2 Nucleation mechanism of ultrasound-assisted electrodeposition of indium

**Fig. 7:**
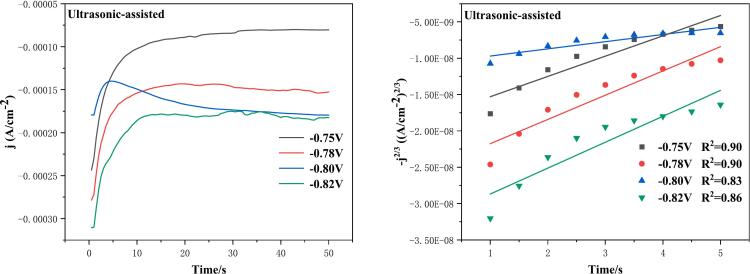
(a) Chronoamperometric curves of ultrasound-assisted electrodeposition of indium at different deposition potentials; (b) −j^2/3^-t plot of the rising part of the transient current; ultrasonic power 100 W.

As illustrated in Fig. 7a, the ultrasound-assisted chronoamperometry curves are shown at varying deposition potentials, with a deposition time of 50 s. In a manner analogous to conventional electrodeposition, chronoamperometric curves demonstrate a comparable tendency, exhibiting an increase in current with time at deposition potentials of −0.75 V, −0.78 V, and −0.82 V, followed by a period of stabilization. However, at the deposition potential of −0.80 V, the chronoamperometric curve increases to a maximum and then decreases gradually, which may be attributed to the introduction of ultrasound accelerating the transfer of indium ions to the electrode, resulting in a decrease in current as indium continues to be deposited on the electrode surface. In order to preliminarily determine the nucleation mode of indium electrodeposited by ultrasound, a linear fit was performed on the rising part of the current and the transient time, thereby obtaining the −j^2/3^-t plot. As demonstrated in Fig. 7b, the linear regression coefficients (R^2^) were 0.83 and 0.86 for deposition potentials of −0.8 V and −0.82 V, respectively. The value of R^2^ was less than 0.9, which was attributed to the failure to remove the anomalous data points during the linear fitting process. This was undertaken to ensure that the data were real and reliable. However, a superior linear relationship was demonstrated in general, which allows for the preliminary inference that the nucleation mode of ultrasonic electrodeposition of indium is progressive nucleation.

**Fig. 8:**
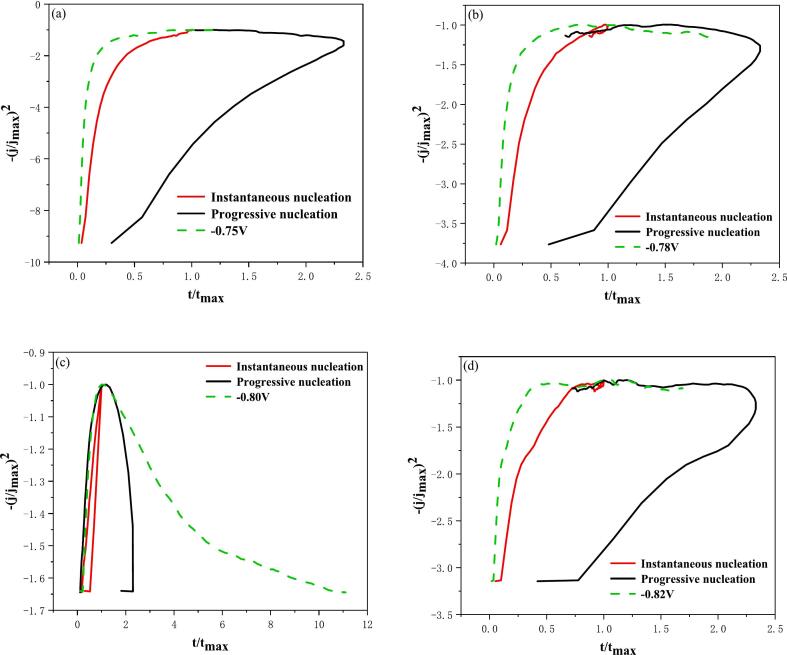
Non-dimensional-(j/j_max_)^2^ vs t/t_max_ plots at different electrodeposition potentials of ultrasound-assisted electrodeposition: (a) −0.75 V; (b) −0.78 V; (c) −0.80 V; (d) −0.82 V.

In a similar manner, the nucleation mode of ultrasound-deposited indium could be more accurately determined by utilizing equations (39), (40) to dimensionless normalize Fig. 7a, thereby yielding Fig. 8.

As demonstrated in Fig. 8, the dimensionless chronoamperometric curves demonstrate a stronger alignment with the theoretical transient nucleation model curves at ultrasound-assisted deposition potentials of −0.75 V, −0.78 V, and −0.82 V. Consequently, the electrodeposition of indium at these ultrasound-assisted deposition potentials can be classified as transient nucleation. It is interesting to note that the dimensionless chronoamperometric curve is more closely aligned with the theoretical curve of the asymptotic nucleation model when the deposition potential is −0.80 V. Consequently, the ultrasound-assisted electrodeposition of indium can be classified as asymptotic nucleation. At a deposition potential of −0.8 V, results show that this nucleation process is more homogeneous and continuous due to the cavitation effect and micro-stirring, facilitating the transition to a progressive nucleation mode at a deposition potential of −0.80 V.

Subsequently, we obtained significant kinetic parameters, including the crystal growth rate (k), diffusion coefficient (D), nucleation rate (K), and nucleation density (N), in accordance with the equations presented in references [38], as illustrated in Table 4. In a similar manner, due to the negative value of the timed amperage curve current, a negative value is placed in front of equation (41) to facilitate calculation.

(41) $$i_{m}=-nFk$$

(42) $$i_{m}^{2}t_{m}=0.1629{\left(nFc\right)}^{2}D$$

(43) $$t_{m}=\frac{1.2564}{N\pi KD}$$

(44) $$K=\sqrt{\frac{8\pi cM}{\rho }}$$

**Table 4:** Kinetic parameters of conventional and ultrasound-assisted electrodeposition chronoamperometric curves at different electrodeposition potentials.

Electrodeposition method	Electrodeposition potential（V）	k (mol cm^−2^ s^−1^)	D (cm^2^/s)	K (s^−1^)	N (cm^−2^)
Conventional electrodeposition	−0.75	5.09◊10^-10^	2.04◊10^-7^	0.08	9.67◊10^8^
	−0.78	8.02◊10^-10^	4.94◊10^-7^	0.08	3.93◊10^8^
	−0.80	7.12◊10^-10^	2.57◊10^-7^	0.08	4.95◊10^8^
	−0.82	7.13◊10^-10^	2.93◊10^-7^	0.08	4.98◊10^8^

Ultrasound-assisted electrodeposition	−0.75	2.78◊10^-10^	5.20◊10^-8^	0.08	3.28◊10^9^
	−0.78	4.96◊10^-10^	8.39◊10^-8^	0.08	1.03◊10^9^
	−0.80	4.84◊10^-10^	1.99◊10^-8^	0.08	1.08◊10^9^
	−0.82	6.06◊10^-10^	2.02◊10^-7^	0.08	6.89◊10^8^

where n is the number of electrons transferred, F is the Faraday constant (96485C/mol), c is the concentration (mol/cm^3^), M is the molar mass (g/mol), ρ is the density (g/cm^3^), K is the nucleation rate, and D is the diffusion coefficient (cm^2^/s).

As demonstrated in Table 4, the nucleation density value (N) of ultrasound-assisted electrodeposition exceeds that of conventional electrodeposition at equivalent electrodeposition potentials. This is attributable to the generation of microjets beneath the cavitation effect of ultrasound, which curtails the proliferation of dendrites during the nucleation process of indium electrodeposition. Consequently, ultrasound effectively promotes indium to attain a uniform, smooth, and dense electrodeposition. At varying deposition potentials, given the relatively brief electrodeposition time, it is hypothesised that the concentration of indium ions in the electrolyte remains constant. Consequently, K is assumed to be constant. However, at varying deposition potentials, the peak current density is subject to alteration, thereby rendering k a variable quantity.

### 3.3 Effect of ultrasonic power on current efficiency

**Fig. 9:**
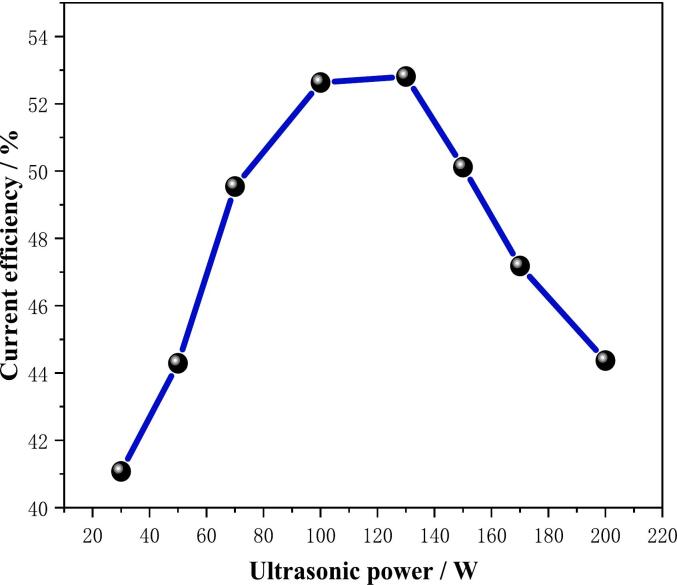
Effect of ultrasonic power on current efficiency (Deposition potential: −0.80 V, Deposition time: 30 min).

The effect of ultrasonic power on current efficiency is illustrated in Fig. 9. It is evident from the figure that as ultrasonic power increases, current efficiency shows a gradual rise. At powers of 100 W and 130 W, current efficiency reaches 52.6 % and 52.8 %, respectively. Subsequently, an increase in ultrasonic power results in a decline in current efficiency, suggesting that elevated ultrasonic power is detrimental to indium electrodeposition. The underlying reason for this phenomenon is believed to be the cavitation effect of high-power ultrasound, which has been shown to significantly increase the local temperature of the electrolyte. This, in turn, has been demonstrated to alter the equilibrium potential of the electrode reaction and to cause a decrease in the overpotential of the hydrogen precipitation reaction. This, in turn, has been shown to lead to an intensification of the hydrogen precipitation reaction and a decrease in the current efficiency. Furthermore, the intense ultrasonic cavitation may result in the formation of numerous minute bubbles on the electrode surface, thereby establishing a local air film. This hinders the mass transfer path of metal ions, reduces the effective reaction area, and consequently leads to a decrease in current efficiency.

### 3.4 Comparison of current efficiency between conventional and ultrasound-assisted electrodeposition

As demonstrated in Table 5, a comparison of current efficiency between conventional electrodeposition and ultrasound-assisted electrodeposition of indium is provided. The results indicate that the current efficiency of the latter is 18.56 percentage points higher than that of the former. This finding suggests that ultrasound can accelerate the movement of indium ions to the cathode, reduce polarisation, lower the deposition overpotential, and consequently enhance current efficiency. This result is consistent with previous findings in which some researchers demonstrated that ultrasound can improve the current efficiency of electrodeposition [39].

**Table 5:** Current efficiency of conventional and ultrasound-assisted electrodeposition.

Electrodeposition method	Electrodeposition potential（V）	Current efficiency (%)
Conventional electrodeposition	−0.80	34.07
Ultrasound-assisted electrodeposition	−0.80	52.63

### 3.5 Mechanism of ultrasound-assisted electrodeposition of indium

**Fig. 10:**
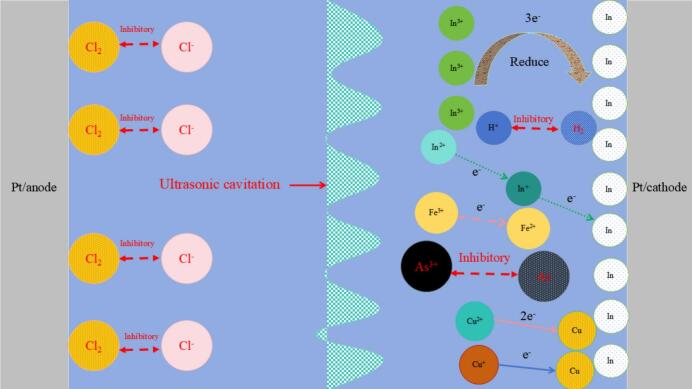
Mechanistic plot of ultrasound assisted electrodeposition of indium.

The CV-EQCM curves of ultrasound-assisted electrodeposition of indium were analyzed, and the mechanism of ultrasound-assisted electrodeposition of indium was plotted (see Fig. 10). As demonstrated in Fig. 10, the valence conversion mechanism of the ultrasound-assisted electrodeposition of indium is In^3+^-In, In^2+^-In^+^-In. However, conventional electrodepositions are In^3+^-In, In^2+^-In^+^. Furthermore, the conventional electrodeposition of indium is accompanied by the reduction of As^3+^ to As at the cathode and the oxidation of Cl^-^ to Cl_2_ at the anode, along with the precipitation of hydrogen, which is unfavorable. However, ultrasound-assisted electrodeposition suppressed the reduction of As^3+^ to As and the oxidation of Cl^-^ to Cl_2_, and hydrogen precipitation was also suppressed. The cavitation effect of ultrasound has been demonstrated to result in the formation of micro-jets in the cathode area, thereby significantly reducing cathode and anode polarization. This, in turn, has been shown to lead to a reduction in hydrogen ions and trivalent arsenic concentration on the cathode surface, as well as a reduction in chloride ions in the anode area. Consequently, ultrasound-assisted has been demonstrated to be an effective means of inhibiting the precipitation of hydrogen and chloride, and of preventing trivalent arsenic from being reduced at the cathode.

### 3.6 Characterization

#### 3.6.1 UV–vis absorption spectroscopy analysis of stripping solutions and electrodeposition residual solutions

**Fig. 11:**
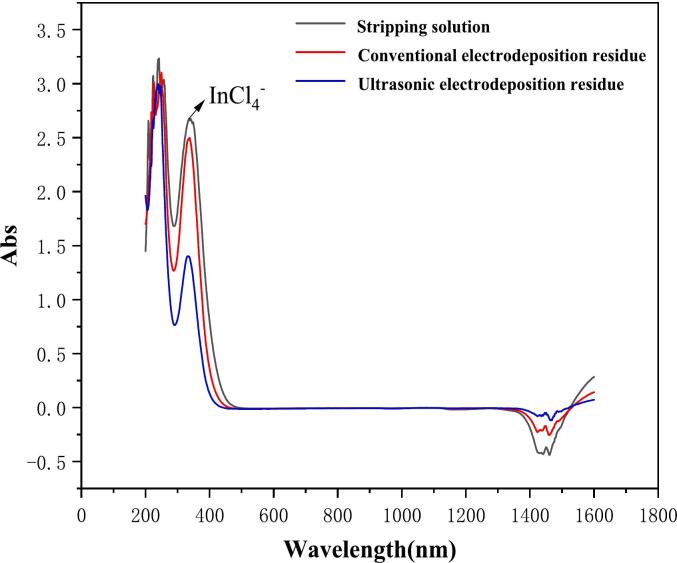
UV–vis of stripping solution, conventional electrodeposition of indium residual solution, ultrasound assisted electrodeposition of indium residual solution (Deposition potential: −0.80 V, Deposition time: 30 min, Temperature 25℃, ultrasonic power 100 W).

As illustrated in Fig. 11, the UV–vis absorption spectroscopy of the stripping solution, conventional electrodeposition of indium, and the residual solution of ultrasound-assisted electrodeposition of indium are presented.

As demonstrated in Fig. 11, the absorption peak intensities of the conventional and ultrasonic electrodeposition residual solutions were diminished in comparison to the stripping solution for an equivalent deposition duration. However, the absorption peak intensity of the ultrasound-assisted electrodeposition residual solution was weaker than that of conventional electrodeposition. This finding suggests that the deposition rate of ultrasound-assisted electrodeposition is faster and that the concentration of indium ions in the ultrasound-assisted electrodeposition residual solution is lower than that of conventional electrodeposition. This finding suggests that the introduction of ultrasound can accelerate the rate of indium deposition.

#### 3.6.2 XRD of sediments

**Fig. 12:**
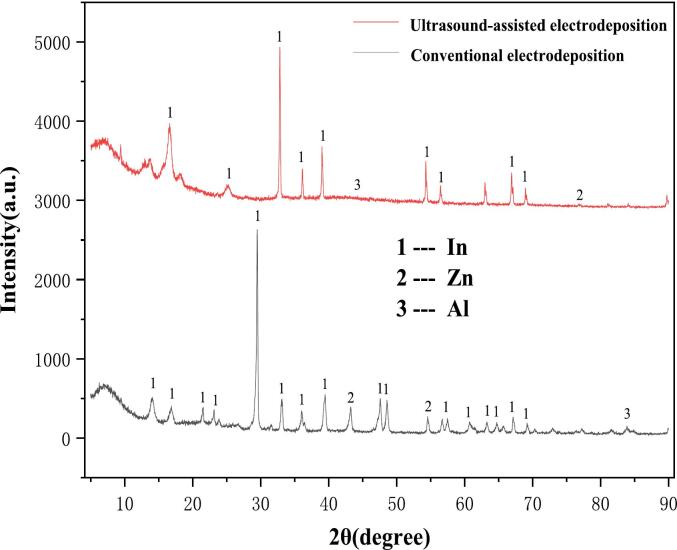
XRD pattern of conventional electrodeposition and ultrasound-assisted electrodeposition (Deposition potential: −0.80 V, Deposition time: 30 min, Temperature 25℃, ultrasonic power 100 W).

As illustrated in Fig. 12, the XRD patterns of the deposited layers of conventionally electrodeposited indium and of indium electrodeposited using ultrasound assistance are shown.

As demonstrated in Fig. 12, the predominant physical phase of the conventionally and ultrasonically deposited layers is indium, accompanied by trace quantities of zinc and aluminum. Furthermore, the diffraction peaks of the physical phases of zinc and aluminum impurities in the deposited layers of ultrasound-assisted electrodeposition are less pronounced than those of conventional electrodeposition, which serves as evidence that ultrasound can inhibit the electrodeposition of zinc and aluminum.

#### 3.6.3 SEM of sediments

**Fig. 13:**
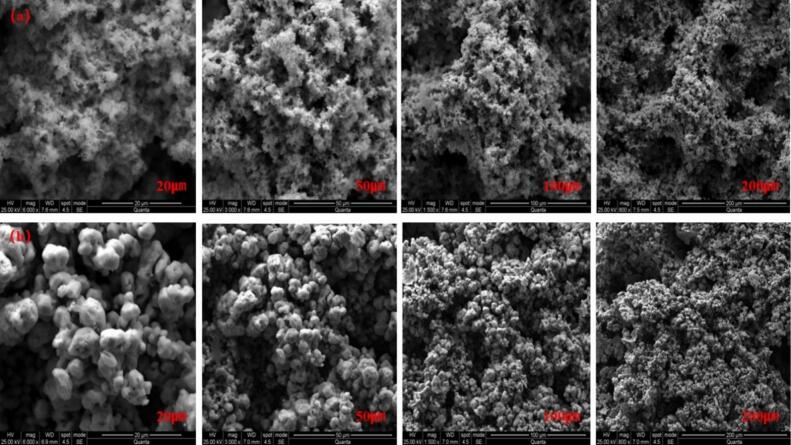
SEM of the deposited layer. a. Conventional electrodeposition; b. Ultrasound-assisted electrodeposition; Deposition potential: −0.80 V, Deposition time: 30 min, Temperature 25℃, ultrasonic power 100 W.

As illustrated in Fig. 13, the surface morphology of indium deposits is observed at varying magnifications for both conventional electrodeposition and ultrasound-assisted electrodeposition under equivalent deposition conditions.

As demonstrated in Fig. 13, the surface morphology of the deposited layer of conventionally electrodeposited indium is predominantly flocculent, characterized by protrusions on the surface and loose grains. The surface morphology of the deposited layer of indium, produced by ultrasound-assisted electrodeposition, is primarily spherical, exhibiting a flat surface and dense grains. It has been demonstrated that the electrodeposition of indium at a deposition potential of −0.80 V, conventional electrodeposition, is a transient nucleation process. The indium deposited on the Pt electrode grows directly into a flocculent shape. In contrast, ultrasound-assisted electrodeposition of indium is a gradual nucleation process, and the indium deposited on the Pt electrode grows into a spherical shape through gradual nucleation. As demonstrated by Zhang et al., the utilization of ultrasound in conjunction with electrodeposition has been shown to result in a deposit layer that is both more uniform and denser in nature [40]. Furthermore, the cavitation of ultrasound results in the generation of bubbles, accompanied by the formation of local high pressure and micro-jet flow. The subsequent bursting of these bubbles gives rise to the formation of spherical structures on the surface of the deposited indium particles. The application of ultrasound has been demonstrated to facilitate the uniform distribution of indium ions within the electrolyte, thereby promoting uniform nucleation and the formation of fine spherical particles. Furthermore, ultrasound has been shown to disrupt the dendritic structures that are growing, thereby inhibiting the extension of the dendrites and promoting the formation of spherical particles.

#### 3.6.4 EDS of sediments

**Fig. 14:**
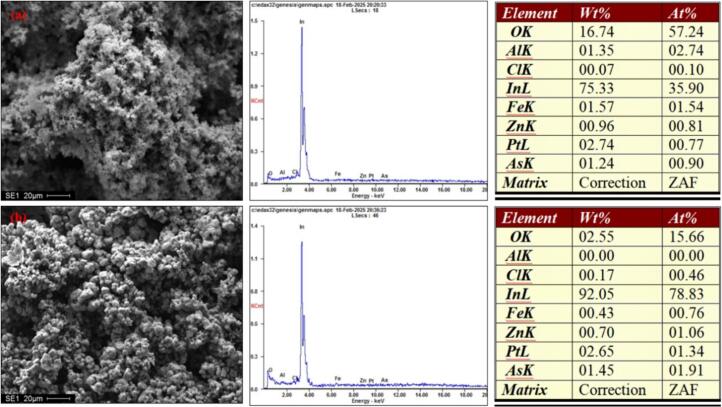
EDS of the deposited layer. a. Conventional electrodeposition; b. Ultrasound-assisted electrodeposition; Deposition potential: −0.80 V, Deposition time: 30 min, Temperature 25℃, ultrasonic power 100 W.

As illustrated in Fig. 14, the energy spectrum of conventionally electrodeposited indium and ultrasonically electrodeposited indium deposited layers is shown at a magnification of 1500.

As demonstrated in Fig. 14, the primary component of the deposition layer in conventional electrodeposition and ultrasound-assisted electrodeposition of indium is monolithic indium, accompanied by a minimal presence of impurities such as zinc, iron, and aluminum, among others. Furthermore, the indium content in the ultrasound-assisted electrodeposition of indium is higher than that of the conventional electrodeposition of indium. This suggests that ultrasound-assisted electrodepositions may promote the electrodeposition of indium. It has been demonstrated that the electrodeposition of impurities, including zinc, iron, aluminum, and arsenic, is suppressed by the cavitation effect of ultrasound.

## 4 Conclusion

The experimental program involved the assessment of cyclic voltammetry-ECQM curves, chronoamperometric curves for conventional electrodeposition and ultrasound-assisted electrodeposition of indium in stripping solutions using an electrochemical quartz crystal microbalance (EQCM). A comprehensive investigative approach was undertaken, encompassing UV–vis analysis of the electrodeposition residual solution, XRD evaluation, and SEM-EDS analysis of the constant potential deposited layer. This multifaceted examination yielded several salient conclusions, which are outlined below:

(1) The valence states of indium in conventional electrodeposition are shifted to In^2+^-In^+^ and In^3+^-In; the valence states of indium in ultrasound-assisted electrodeposition are shifted to In^2+^-In^+^-In and In^3+^-In.

(2) The ultrasound-assisted electrodeposition process has been shown to prevent hydrogen, chlorine and arsenic from precipitating at the cathode.

(3) In view of the negative current value of the timed amperage curve, the Scharifker and Hills model was modified to ensure that the dimensionless processed timed amperage curve (j/j_max_)^2^-(t/t_max_) is in compliance with the specification.

(4) The conventional electrodeposition process is characterized by transient nucleation at deposition potentials of −0.75 V, −0.78 V, −0.80 V, and −0.82 V. In contrast, the ultrasound-assisted electrodeposition process exhibits transient nucleation at deposition potentials of −0.75 V, −0.78 V, and −0.82 V, with gradual nucleation occurring at the −0.80 V potential. Furthermore, at the same electrodeposition potential, ultrasound-assisted electrodeposition resulted in greater nucleation density values (N) than conventional electrodeposition.

(5) It has been demonstrated that ultrasound-assisted electrodeposition has the capacity to enhance current efficiency. In addition, SEM-EDS analysis has revealed that the indium deposited on the cathode is of high purity. Furthermore, the surface morphology of the indium layer deposited by conventional electrodeposition has been observed to be flocculent, while that of the indium layer deposited by ultrasound-assisted electrodeposition has been shown to be spherical.

The ultrasound-assisted direct electrodeposition of indium from stripping solution offers a novel approach to the short-flow recovery process of indium. Future research should concentrate on the control of chlorine and hydrogen precipitation, which can be utilized in the synthesis of hydrochloric acid, thereby rendering the process more environmentally friendly and economically.

## CRediT authorship contribution statement

**Shiju Li:** Investigation, Data curation, Writing – original draft. **Haibei Wang:** Supervision, Resources, Project administration, Methodology, Funding acquisition. **Shengdong Wang:** Software, Resources. **Feng Xie:** Project administration. **Xudong Sun:** Software.

## Declaration of competing interest

The authors declare that they have no known competing financial interests or personal relationships that could have appeared to influence the work reported in this paper.
